# Statistics and behavior of clinically significant extra-pulmonary vein atrial fibrillation sources: machine-learning-enhanced electrographic flow mapping in persistent atrial fibrillation

**DOI:** 10.3389/fcvm.2025.1517484

**Published:** 2025-08-26

**Authors:** Peter Ruppersberg, Steven Castellano, Philip Haeusser, Kostiantyn Ahapov, Melissa H. Kong, Stefan G. Spitzer, Georg Nölker, Andreas Rillig, Tamas Szili-Torok

**Affiliations:** ^1^ Cortex, Inc. Menlo Park, CA, United States; ^2^ Praxisklinik Herz und Gefäße Dresden, Akademische Lehrpraxisklinik der TU Dresden, Dresden, Germany; ^3^ Brandenburg University of Technology Cottbus-Senftenberg, Institute of Medical Technology, Cottbus, Germany; ^4^ Department of Cardiology, Heart and Diabetes Center North Rhine-Westphalia, Ruhr University Bochum, Bad Oyenhausen, Germany; ^5^ Interventional Electrophysiology, University Heart Center, Hamburg, Germany; ^6^ Department of Cardiology, University of Szeged Albert Szent-Györgyi Medical School, Szeged, Hungary

**Keywords:** persistent atrial fibrillation, electrographic flow mapping, basket catheter, panoramic mapping, machine learning, clinical validation

## Abstract

**Introduction:**

Electrographic flow (EGF) mapping is an FDA 510(k)-cleared method for visualizing atrial activation wavefronts in atrial fibrillation (AF). Its clinical efficacy was demonstrated in the FLOW-AF randomized controlled trial, and its fundamental principles have been previously described. However, the underlying machine learning strategy used to develop and refine the EGF algorithm has not yet been detailed. Here, we present how our EGF Model—trained on procedural outcomes from 199 fully anonymized retrospective patient datasets—identifies clinically significant sources of AF and how this machine learning–driven hyperparameter optimization underlies its clinical effectiveness. We also examine the statistical characteristics of the identified sources and their impact on cycle length variability, offering insights into potential pathophysiological mechanisms.

**Methods and results:**

Unipolar electrograms were recorded from patients with persistent or long-standing persistent AF using 64-electrode basket catheters. The EGF Model processes these recordings to reconstruct divergent wavefront propagation patterns and quantify their temporal prevalence. We included 399 retrospective patients in total: 199 for training and optimizing 24 model hyperparameters, and 200 for subsequent analyses of source prevalence and characteristics. Our machine learning approach established an activity threshold, above which divergent wavefront patterns—termed “significant sources”—predicted AF recurrence. This threshold was validated in 85 prospective patients from the published FLOW-AF trial. Significant sources persisting post-procedure were associated with significantly higher recurrence rates than those successfully ablated. Notably, the majority of significant sources were not continuously active; however, when these sources switched “ON,” the spatial variability of AF cycle lengths in the respective atrium decreased by more than 50%, suggesting an entraining effect.

**Conclusions:**

By systematically optimizing the EGF Model's hyperparameters based on clinical outcomes, we reliably detect and target key AF sources that, when ablated, improve procedural success. These findings, supported by the FLOW-AF trial, underscore the usefulness of clinical outcome-based machine learning to improve the efficacy of algorithm based medical diagnostics.

## Introduction

Atrial fibrillation (AF) is characterized by chaotic and complex atrial activations. While pulmonary vein (PV) triggers remain the most widely recognized initiators of AF (1), many patients with persistent or long-standing persistent AF show evidence of extra-PV mechanisms (2–4). These mechanisms may include focal firing sites (3), micro-reentry (5), macro-reentry (6), or epicardial-to-endocardial breakthroughs (7, 8), though their precise roles in sustaining AF continue to be debated (9, 10).

Efforts to localize and ablate AF-maintaining drivers have produced limited improvements in outcomes (6, 11–15), underscoring the difficulty of identifying truly actionable sources. Electrographic flow (EGF) mapping offers a novel approach by reconstructing and analyzing time series of divergent wavefront propagation vectors derived from 64-electrode basket recordings (16–24). This EGF method has demonstrated meaningful clinical benefit in the FLOW-AF randomized controlled trial (22), and its foundational principles have been reported in prior work. However, the published studies have not focused on the machine learning strategy based on retrospective patient data that was originally used to optimize the EGF Model's hyperparameters—the critical training process for enabling the algorithm to detect clinically significant sources that, once ablated, may improve outcomes. Unlike randomly seeded neural network training approaches, which require hundreds of thousands of patient datasets to learn both the underlying physiology and its correlation with outcomes (25), the EGF approach began with a mechanistic model for divergent wavefront propagation. This mechanistic grounding reduced the number of free parameters and thereby the data requirement for training while still representing a trainable model capable of identifying clinically significant AF sources.

In this paper, we describe the development and hyperparameter optimization of the EGF Model. We detail how 24 hyperparameters were trained on procedural outcomes in a cohort of 199 anonymized retrospective patient datasets with persistent or long-standing persistent AF for which 12-month outcomes were available. We then present statistical characterizations of AF sources in the total set of 399 retrospective patients. Finally, we refer to the independent validation of these hyperparameter settings in the published FLOW-AF trial, involving 85 prospective cases. Taken together, these complementary datasets demonstrate how a machine learning–optimized EGF mapping approach can accurately pinpoint meaningful ablation targets and thereby improve clinical outcomes.

## Methods

### Study population and data sources

This study retrospectively analyzed anonymized datasets from 399 patients with persistent or long-standing persistent atrial fibrillation (AF) who underwent ablation procedures using 64-electrode basket catheters across five European centers (Erasmus Medical Center, Netherlands; Praxisklinik Herz und Gefäße, Dresden, Germany; Ruhr University, Bad Oeynhausen, Germany; Asklepios Clinic St. Georg, Hamburg, Germany; and Charité, Benjamin Franklin Clinic, Berlin, Germany). Procedures were performed under sedation with systemic anticoagulation, transseptal catheterization, and 3D electroanatomic mapping (Carto or EnSite Precision). A total of 62% had previously undergone AF ablation.

From this pool, 199 patients were specifically selected for training and testing the Electrographic Flow (EGF) Model's hyperparameters. Criteria for inclusion were availability of final intra-procedural recordings from both atria, documented 12-month clinical outcomes, and acceptable signal quality with minimal baseline drift. The remaining 200 patients served to characterize and analyze statistical properties and prevalence of AF sources as identified by the fully optimized EGF Model, including their impact on atrial cycle length variability.

### EGF mapping: basic principles

The EGF Model, a core component of OptiMap® software (Cortex Inc., Santa Clara, CA, USA), processes 1 min unipolar electrogram recordings sampled at 1 kHz from basket catheters placed in both atria. Signals undergo preprocessing, including noise reduction, normalization, and removal of far-field ventricular activity. Local activation vectors are computed using a modified Horn–Schunck optical flow algorithm (see Supplementary Material for details).

**Figure 1:**
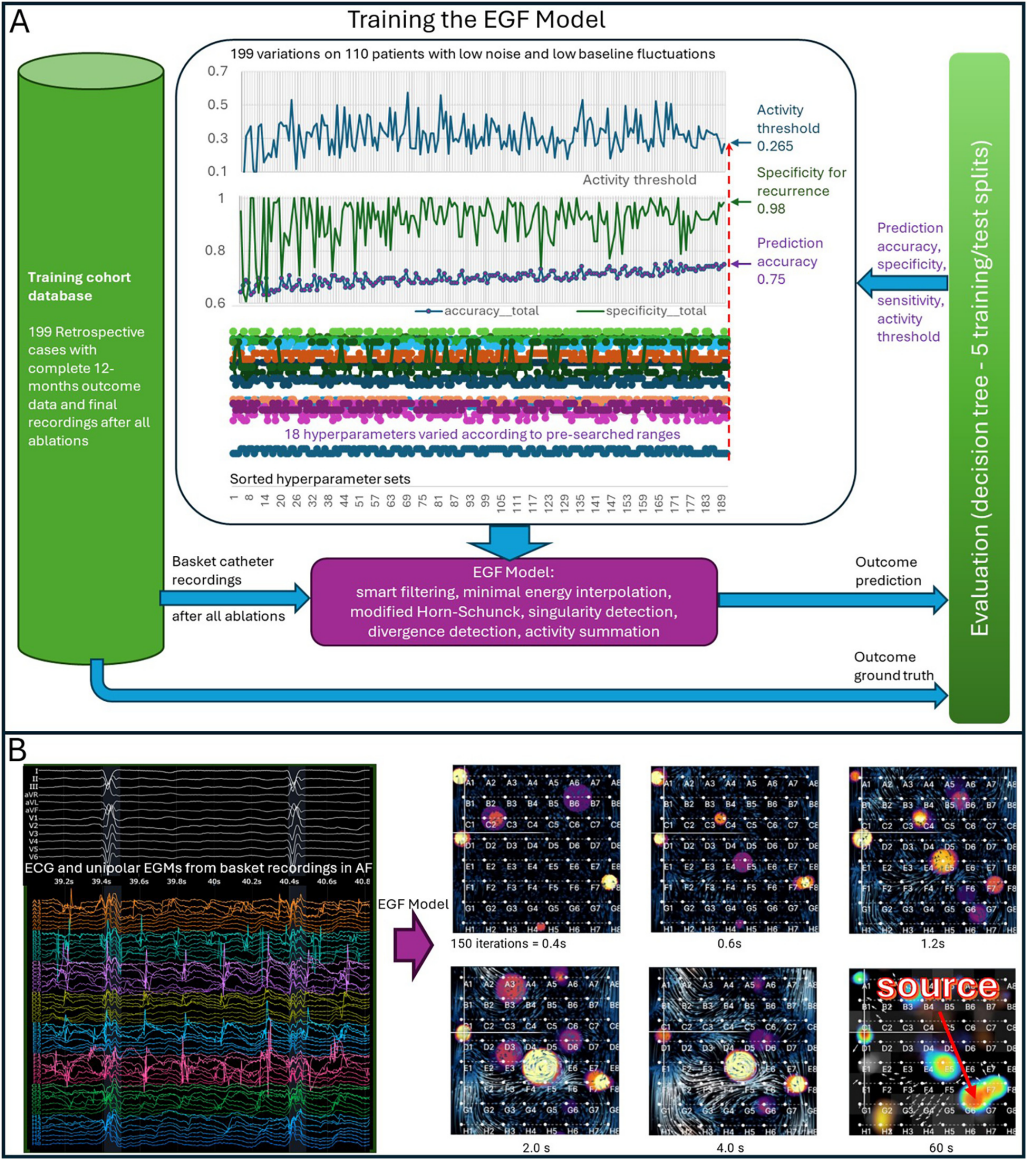
Training the EGF model and evolution of an electrographic flow (EGF) Map. **(A)** Hyperparameter training of the EGF Model was conducted in iterative cycles using different subgroups of the 199-patient training cohort. Each step on the *x*-axis represents one cross-validation cycle involving 110 patients. The top panel shows the progression of the activity threshold (cutoff) over these cycles. The middle panel displays specificity (i.e., ability to avoid false positives) and overall prediction accuracy. The bottom panel illustrates the ranges tested for 18 hyperparameters and how they converged to the final optimized settings. **(B)** Evolution of AF sources in an EGF map. **Left**: A representative 64-electrode basket recording. **Right**: After 0.4 s (150 EGF iterations), six source candidates begin to emerge. As time progresses and more frames accumulate, these sources stabilize. By 60 s, three distinct sources remain, one of which is dominant. The 60-second EGF Summary Map, shown as a heat map, highlights the spatial distribution and prevalence of these sources.

**Figure 2:**
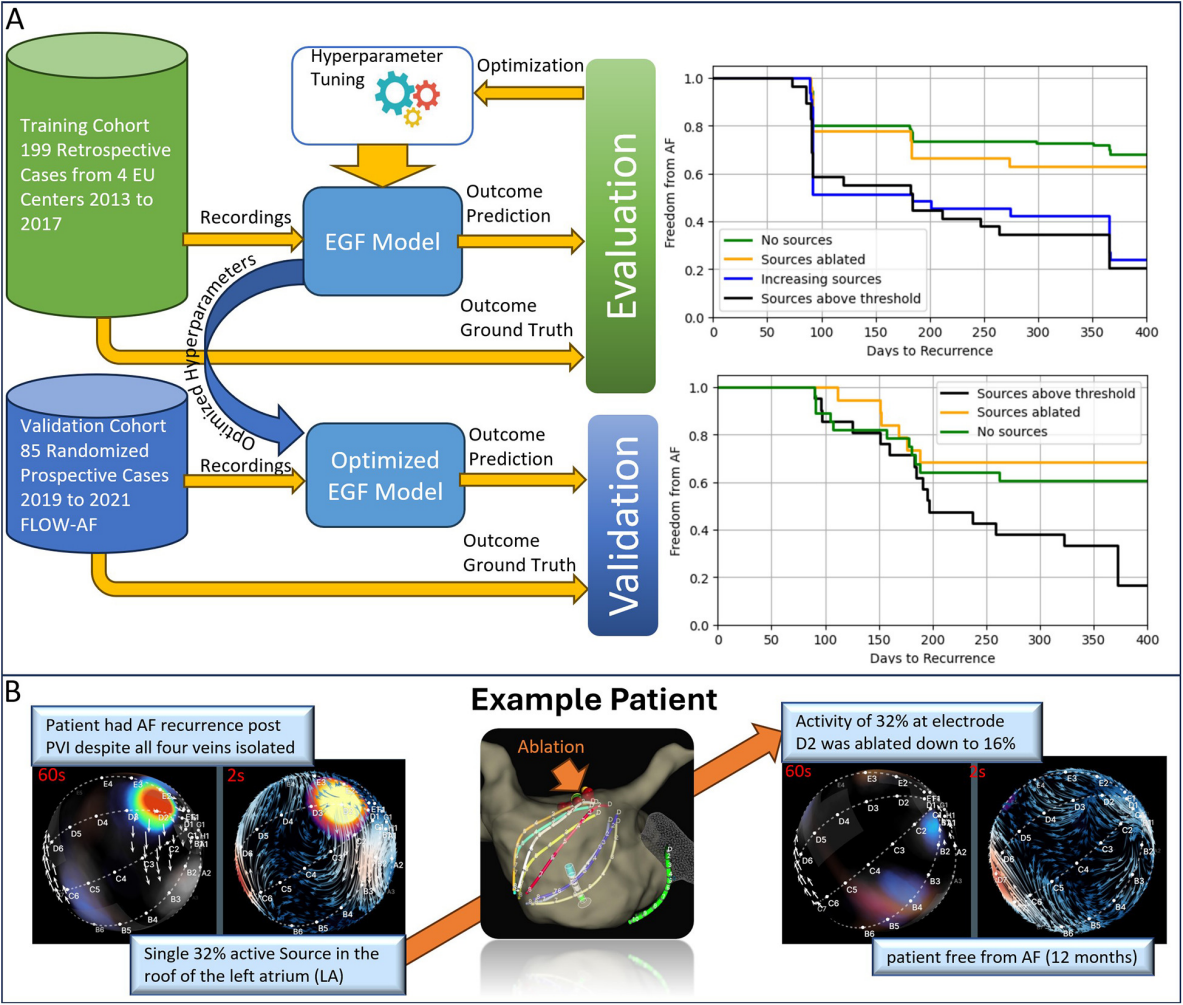
Validation of the EGF model for source ablation. **(A)** The top panel shows results from the training cohort of 199 retrospective cases collected from four European centers (2013–2017). A 42% difference in freedom from AF was observed between patients whose final ablation recordings showed sources ≥26.5% activity vs. those below this threshold. The bottom panel presents data from 85 patients in the FLOW-AF randomized prospective trial (2019–2021). Here, a 51% improvement in one-year AF-free survival is seen in patients who underwent EGF-guided source ablation compared to PVI-only. **(B)** An example case of a patient with recurrent AF following an initial PVI. EGF mapping identified a single, clinically significant source (32% activity) in the LA, consistently reproduced across three consecutive 60 s recordings. After ablating this source and performing cardioversion, the patient remained AF-free at 12 months.

Wavefront Reconstruction involves combining these vectors over consecutive, overlapping 2-second segments to identify statistically dominant wavefront propagation patterns. Divergent wavefronts originating from local singularities, termed “sources,” are tracked throughout each recording. Figure 1B illustrates the evolution of these source candidates, which begin to crystallize after the first 0.4 s. After 4 s, the heatmap represents source prevalence across segments. For each 60 s recording, these analyses are summarized in a Summary Map highlighting potential source regions and their temporal prevalence (see Figures 1, 2).

### AF source detection, prevalence, and activity

After training, each 60 s recording was analyzed to identify atrial sources. Prevalence was defined as the percentage of recording time a specific source was detected. Sources were termed “dominant” if their prevalence exceeded 20%. Activity, the average prevalence per 2 s segment normalized to the source's maximum prevalence, adjusted for electrode contact or noise artifacts. A clinically significant activity threshold of 26.5%, established during hyperparameter optimization, reliably predicted AF recurrence.

### Cycle length analysis

Cycle length (CL) was computed via autocorrelation analysis of electrograms, identifying peak intervals that represent local atrial activation. Electrodes yielding autocorrelation below 0.6 or near-field amplitudes below 0.7 times the far-field QRST amplitude were excluded. CL values from remaining electrodes were averaged into 2-second segments, computing mean CL and standard deviation (SD) to assess how dominant sources influenced local atrial activation (e.g., spatial CL variability reduction). Detailed Software and Hyperparameter Description.

A comprehensive technical description of the EGF algorithm, including its theoretical foundations, mathematical formulations, and explicit definitions of all hyperparameters and their optimization procedures, is provided in Supplementary Material.

## Results

### Patient population

This study included 399 patients with persistent or long-standing persistent AF who underwent intra-atrial 64-electrode basket recordings. The average age of the cohort was 63.4 ± 9.2 years, and 41% of participants were female. Mean AF duration prior to mapping was 51 ± 42 months, with 62% having undergone prior PVI and 63% having been treated with at least one class I or III antiarrhythmic drug. The mean short-axis LA diameter was 50 ± 8 mm, and the mean CHA_2_DS_2_-VASc score was 2.2 ± 1.4 (Table 1).

**Table 1:** Baseline demographics.

Characteristic	Total	No dominant source (prevalence <20%)	Dominant source (prevalence ≥20%)	*P*-value
Number of patients, *n*	399^a^	217^a^	182^a^	
Age (years) (mean, SD)	63.4 ± 9.2	62.4 ± 8.8	64.6 ± 9.6	0.005
Sex (Female), *n* (%)	132 (41.4)	58 (33.7)	74 (50.3)	0.003
Body mass index, (mean, SD)	29.1 ± 5.2	29.6 ± 5.6	28.6 ± 4.6	0.23
Left ventricle ejection fraction (mean, SD)	0.57 ± 0.08	0.57 ± 0.08	0.57 ± 0.08	0.77
Left atrial size (mm) (mean, SD)	50.5 ± 7.5	51.8 ± 7.2	48.8 ± 7.4	0.001
AF duration (months) (mean, SD)	51.3 ± 41.5	53.9 ± 42.8	48.2 ± 39.9	0.23
Prior AF ablation, *n* (%)	194 (62.2)	120 (70.6)	74 (51.7)	0.001
Prior antiarrhythmic drug use, *n* (%)	168 (63.4)	91 (60.7)	77 (67.0)	0.08
Hypertension, *n* (%)	204 (73.6)	116 (77.9)	88 (68.8)	0.10
Diabetes mellitus, *n* (%)	45 (18.5)	26 (18.8)	19 (18.1)	1.0
History of CVA/TIA, *n* (%)	22 (9.1)	12 (8.8)	10 (9.3)	1.0
Coronary or vascular disease, *n* (%)	52 (28.1)	27 (25.0)	25 (32.5)	0.32
CHA_2_DS_2_-VASc-score + (mean, SD)^b^	2.2 ± 1.4	2.0 ± 1.3	2.5 ± 1.5	0.004

^a^ Total number of patients analyzed for source activity level; however, for each of these patients, not all baseline demographic variables were available.

^b^ CHA_2_DS_2_-VASc-score is a calculated risk stratification score to predict risk of stroke in AF patients.

AF, atrial fibrillation; CVA/TIA, cerebrovascular accident/transient ischemic attack; SD, standard deviation.

### Training the EGF model to predict clinically significant sources

Of the 399 retrospective patient datasets, 199 were used to optimize the EGF algorithm based on 12-month ablation outcomes. By correlating the presence or absence of post-ablation recurrence with specific EGF-derived flow patterns the algorithm learned to recognize “clinically significant” sources (i.e., sources correlating with AF recurrence). These sources were operationally defined by an activity threshold such that divergent wavefronts occurring with a frequency above this threshold predicted a higher likelihood of recurrence.

A Kaplan–Meier (KM) analysis demonstrated that patients with final EGF-derived source activity ≥26.5% had significantly worse outcomes than those with <26.5% (Table 2, Figure 2A, upper panel). Importantly, patients without a significant source (i.e., below the threshold) had low recurrence rates, matching patients whose previously high-activity sources were effectively ablated. By contrast, patients whose ablation inadvertently led to a new EGF source or who retained a high-activity source showed recurrence rates similar to those who started with a significant source. These findings suggest that sources identified by the optimized EGF Model are not mere epiphenomena of a diseased atrium but rather have a direct causal role in sustaining AF.

**Table 2:** Subgroup statistics of the retrospective training data using the *Z*-test statistics.

Comparison retrospective training data	Z-score	*P*-value
No sources vs. Sources ablated	0.517	0.604
No sources vs. Increasing sources	4.486	<0.001
No sources vs. Sources above threshold	4.613	<0.001
Sources ablated vs. Increasing sources	3.026	0.00247
Sources ablated vs. Sources above threshold	3.213	0.00131
Increasing sources vs. Sources above threshold	0.333	0.738

Number of cases: No sources (*n* = 110), Sources ablated (*n* = 27), Sources with Increasing activity during procedure (*n* = 33), Sources from beginning above threshold (*n* = 29).

### Validation with the FLOW-AF randomized clinical trial

To further confirm the predictive utility of the optimized EGF Model, we referenced 85 randomized prospective cases from the published FLOW-AF trial (21, 22) (NCT04473963). In that study, patients who had undergone PVI and were found to have EGF sources ≥26.5% activity were randomized to either receive no additional treatment or to undergo targeted ablation of these sources. Kaplan–Meier curves revealed a 51% improved outcome at 12 months in the group that received EGF-guided ablation (Figure 2A, lower panel). Patients who retained a source above the threshold experienced higher recurrence rates than those without such a source or those whose sources had been ablated (Table 3). Notably, patients without a significant source at the outset exhibited similarly low recurrence rates as those who underwent successful EGF-guided source ablation, demonstrating the algorithm's practical utility.

**Table 3:** Subgroup statistics from FLOW-AF using the *Z*-test statistics.

Comparison prospective validation data	Z-score	*P*-value
Sources ablated vs. Sources above threshold	2.000	0.0450
No sources vs. Sources above threshold	2.410	0.0160

Number of cases: No sources (*n* = 28), Sources ablated (*n* = 19), Sources from beginning above threshold (*n* = 21).

An illustrative case is shown in Figure 2B. A female patient from the FLOW-AF study presented with persistent AF despite having undergone a recent PVI. Confirmation showed all pulmonary veins were still isolated, a scenario in which additional linear or posterior wall ablation might typically be performed. Instead, the EGF Model identified a single, significant source in the LA roof. Ablation of this site led to freedom from AF over 12 months without further linear ablation, underscoring the effectiveness of using EGF to guide a more targeted approach.

### Prevalence and behavior of sources

**Figure 3:**
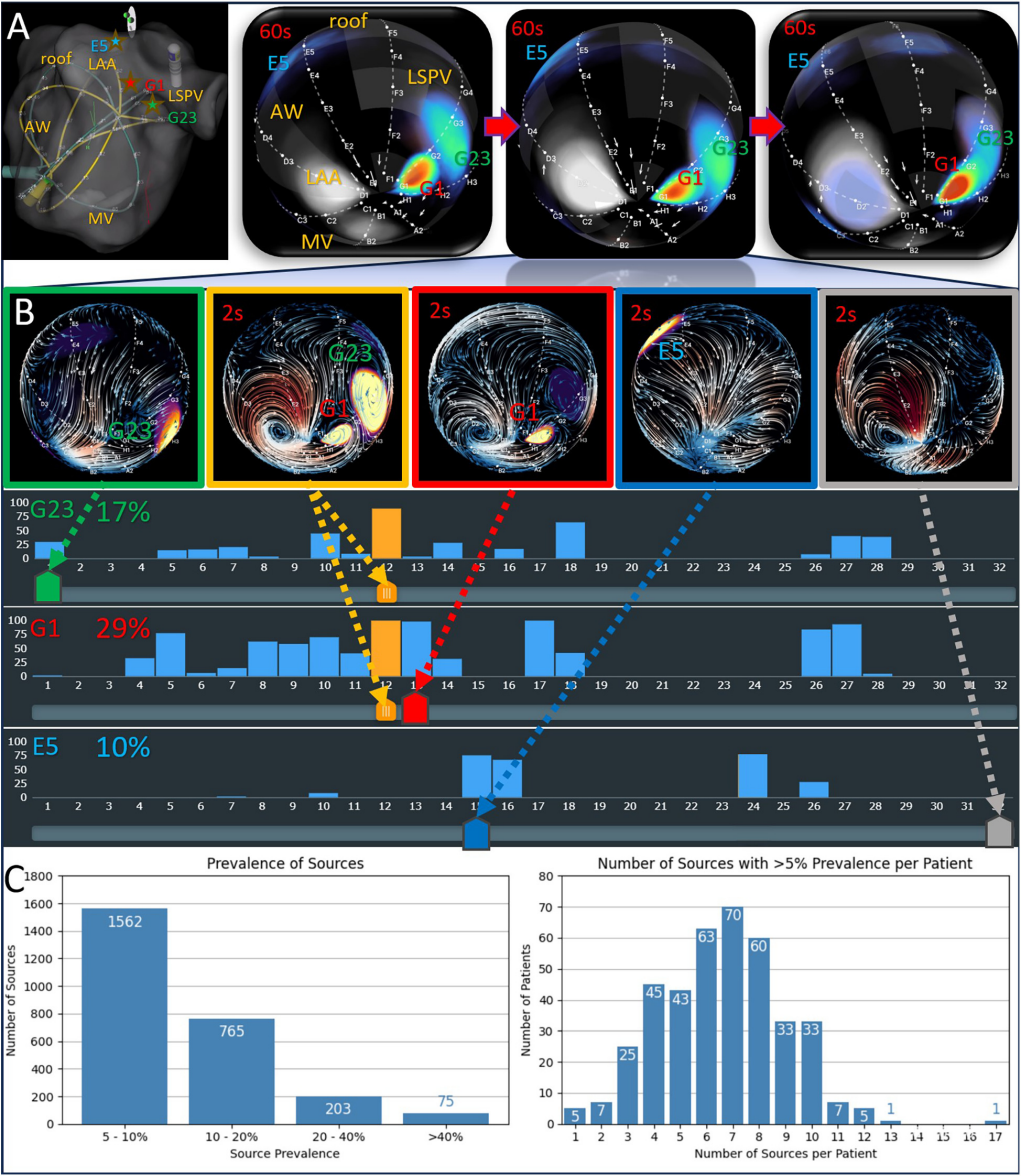
EGF source behavior and prevalence. **(A)** The upper-right three panels show consecutive 60 s recordings made from a central LA basket position (upper-left panel). Recurrent sources at G1 and G23, as well as a weaker source at DE45, appear across these recordings. **(B)** Several 2-second segments depict different activation states: G1 only, G23 only, E5 only, G1 plus G23, and no active sources. The three histograms (corresponding to G1, G23, and E5) display the temporal prevalence of each source during the second 60 s recording. **(C)** Histograms provide an overview of all sources found in the initial recordings (both atria) across the entire patient cohort.

Beyond validating the algorithm, we analyzed the properties of EGF-detected sources across the entire 399-patient cohort. In many cases, multiple sources were observed in a single patient, and these sources often exhibited dynamic “ON/OFF” behavior over each 60 s mapping interval. As shown in Figure 3A, one representative example features three LA sources with measured activities of 29%, 17%, and 10%, corresponding to prevalences of 29%, 15%, and 8%, respectively, in the second of three consecutive 60 s recordings. Despite fluctuating, these three sources appeared consistently in all three consecutive recordings, suggesting reproducible patterns of activation within the expected statistical scatter of their random switching as previously published (23, 24). Only the source with 29% activity met the threshold for a “significant source.”

To further illustrate this dynamic behavior, Figure 3B displays 2-second snippets of each source's activation state, along with histograms quantifying their time-varying prevalence. These histograms reveal periods when two or even all three sources were concurrently active, as well as intervals with no detectable sources. Overall, the case exemplifies how EGF mapping can capture the transient and overlapping nature of multiple AF sources within a short timeframe.

Out of a total of 2,625 sources detected across all initial LA and RA recordings, only 278 (10.7%) met the criteria for dominance (≥20% prevalence). These dominant sources were found in 182 patients (45.7%), yielding an average of 1.5 dominant sources per patient among those harboring them. Geographically, dominant sources were nearly evenly split between the LA (55%) and RA (45%). Patients frequently had several sources with prevalence <20%; the number of such “sub-dominant” sources ranged from 1 to 17 per patient (mean 6.6 ± 2.4).

Interestingly, patients harboring at least one dominant source were older (*p* = 0.005), more likely female (*p* = 0.003), had a smaller LA diameter (*p* < 0.001), and had a higher likelihood of prior AF ablation (*p* = 0.001) than those without any dominant sources (Table 1). The EGF Model design accommodates both focal and rotational wavefronts; the current study showed that about 15% of dominant sources were rotational, indicated by a curl ≥0.7. There were no significant differences in outcomes or average activity between active rotational vs. focal sources (data not shown).

### Significant sources entrain their environment

**Figure 4:**
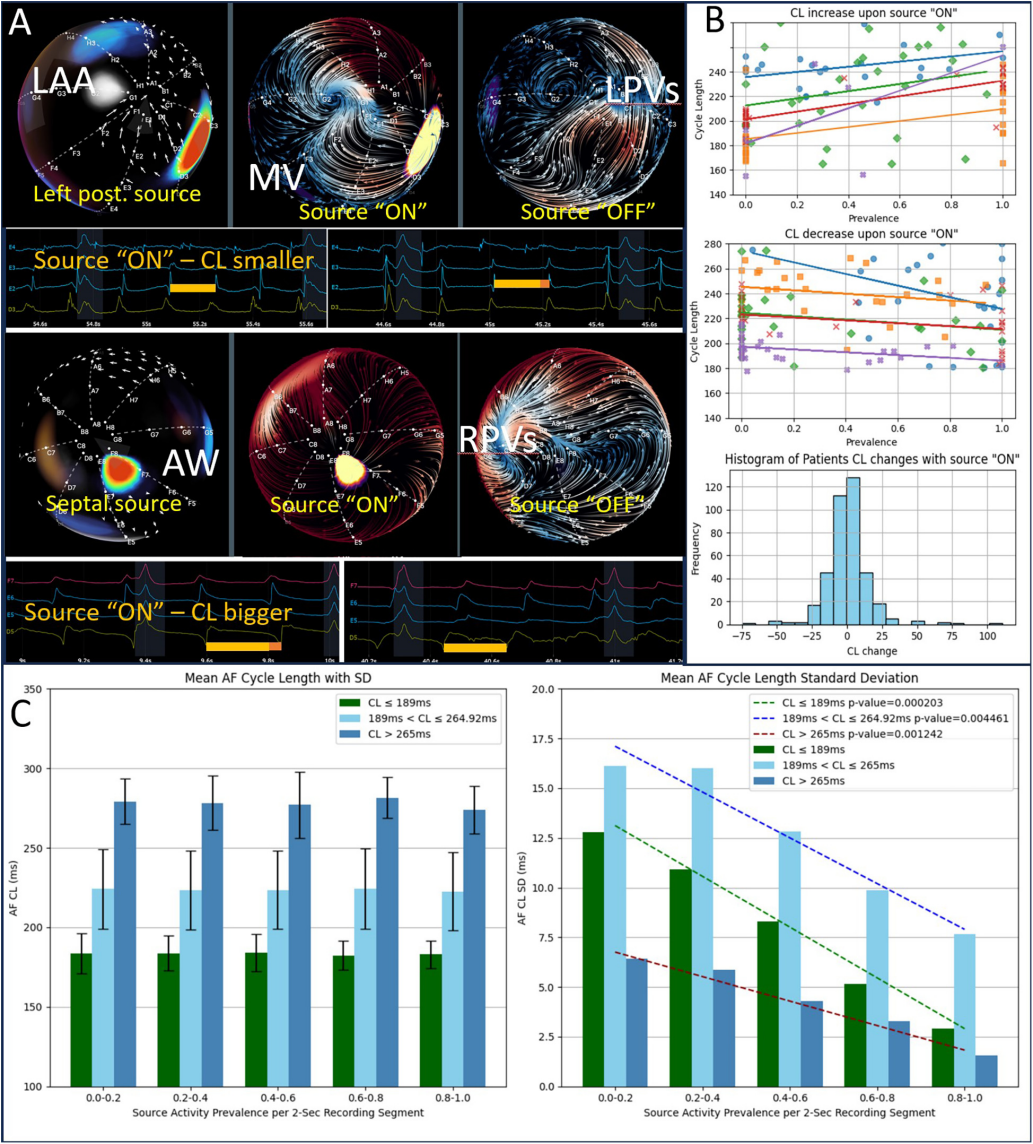
Sources entrain their AF environment. **(A)** Two examples showing changes in cycle length (CL) when a leading source switches “ON.” The top example shows a decrease in CL; the bottom shows an increase. **(B)** The first two panels each summarize five cases where CL increases upon source activation and five cases where CL decreases, respectively, plotted against the source's segment prevalence. The third panel illustrates that, overall, CL changes show a symmetric distribution about zero, indicating no consistent directional effect on the absolute CL. **(C)** The left panel demonstrates that mean CL does not systematically depend on source prevalence, illustrated by grouping patients into high, intermediate, and low baseline CL. However, the right panel shows that the standard deviation of CL (i.e., spatial variability among the basket electrodes) decreases significantly as source prevalence increases in all three groups, indicating enhanced local entrainment when the source is “ON.”

To investigate whether dominant sources influence overall AF dynamics, we examined cycle length (CL) changes upon “ON” switching of these sources. As shown in Figures 4A,B, some sources increased local CL, while others decreased it, suggesting no consistent direction of source effect on dominant frequency (DF = 1/CL) unlike previously presumed (2). As shown in Figure 4C (left panel) that was true for either high CL (*n* = 32), low CL (*n* = 33), or intermediate CL (*n* = 329). However, Figure 4C (right panel) demonstrates that when a dominant source was active, the spatial standard deviation of CL among all recording electrodes diminished by two- to three-fold for all three groups of CL. This suggests that while sources do not universally affect mean CL in a single direction, they appear to entrain nearby tissue, creating more uniform cycle lengths and, thus, more organized AF.

Together, these results reinforce the concept of EGF-detected dominant sources as pivotal organizing sites for AF. By identifying and targeting these sources via ablation, clinicians may significantly improve long-term outcomes in patients with persistent or long-standing persistent AF.

## Discussion

### Extra-PV sources

Long-term results of PVI-only catheter ablation in persistent AF remain suboptimal (26, 27). Increasing evidence suggests that extra-PV drivers and triggers can sustain AF in many patients (4, 13, 28, 29, 30), motivating diverse strategies to detect and eliminate these sites (31, 32).

Unfortunately, most current source-detection algorithms based on mechanistic models have not substantially improved clinical outcomes (6, 11–14, 33). One recent trial used an AI-trained algorithm targeting spatiotemporal dispersion and achieved improved outcomes. However, that approach was trained on experts' visual interpretation rather than actual procedural outcomes, leading to high sensitivity but low specificity for clinically significant sources. Consequently, it sometimes guided more extensive ablation outside the pulmonary veins (34), even in patients who may otherwise have been adequately treated by PVI alone.

### EGF mapping with machine learning based on procedural outcome

In the present study, we describe a new machine learning strategy in which the EGF Model's hyperparameters are optimized based on procedural outcomes rather than human labeling. By integrating a highly parameterized mechanistic model with outcome-driven adjustments, we identified clinically significant sources with high specificity. Despite the inherent spatial limitations of low-density basket catheters, the trained EGF Model effectively pinpoints actionable AF sources. Besides divergent wavefront patterns there are also other output parameters that can be obtained from EGF maps such as EGFC the activation wavefront flow consistency which was recognized as useful to identify patients who respond poorly overall (22). EGFC was not part of the hyperparameter optimization, however, because it was not yet systematically measured at the time. In FLOW-AF, patients with advanced remodeling or low EGFC did not benefit as much from the EGF ablation strategy, consistent with known challenges in treating long-standing persistent AF.

A key advantage of using a mechanistic model-based algorithm, such as the EGF Model, is that it requires far fewer training datasets compared to purely data-driven neural network approaches. Because the EGF algorithm is anchored to well-established physiological principles—namely, that atrial activation wavefronts originate from a singular focal or rotational source—the model does not have to re-learn these fundamentals from scratch. In contrast, a neural network starting from randomized parameters typically needs exceptionally large datasets to capture both the basic electrophysiological rules of AF and their relationship to clinical outcomes. For example, the groundbreaking study from Mayo Clinic trained neural networks on more than 180,000 patients and nearly 650,000 sinus-rhythm ECGs to achieve robust performance in predicting AF occurrence (25). In our setting, by focusing on divergent wavefronts and integrating real-world outcome data from 199 retrospective patients, we were able to systematically refine the EGF algorithm's hyperparameters to pinpoint clinically significant sources with high specificity—even with a comparatively modest training cohort.

### Source behavior and AF cycle length correlation

Dominant sources appear in 182 of 399 patients. Among these patients, we found on average only 1.5 dominant sources per patient, distributed widely throughout both atria (19). In contrast, the average number of subdominant (lower-prevalence) sources was much higher (6.6 per patient). This is noteworthy because previous mapping technologies often identified substantially more targets to ablate (6, 12, 15, 28, 31), indicating that our outcome-based machine learning approach can streamline and minimize ablation by focusing only on those sources most likely to sustain AF.

Once a dominant source switched “ON,” surrounding cycle length variability was substantially reduced, suggesting that these sources entrain local tissue. Our previous experimental work showed that an artificial source induced by pacing can immediately supplant native sources in the EGF map, with the original sources reemerging only after pacing is halted (35). By contrast, other mapping methods failed to detect pacing sites as sources and have sometimes identified epiphenomenal rotations or focal breakthroughs unrelated to AF induction or maintenance, failing to detect the true driver sites (36).

## Study limitations

Despite the encouraging results and rigorous methodology, this study has several limitations.

While our analysis is supported by a substantial retrospective dataset (399 patients in total), these data were anonymized, limiting the amount of detailed clinical information (e.g., precise AF onset dates, presence or absence of sinus rhythm at arrival in the lab, etc.) that could be collected.

The EGF Model relies on data from 64-electrode basket catheters, which inherently have a lower spatial resolution than high-density mapping systems. Although we show that a mechanistic, outcome-based machine learning approach can overcome some of these limitations, the findings may not directly translate to other mapping modalities or catheter designs without further validation.

Electrographic flow consistency (EGFC) was not systematically measured in all retrospective data used to train the EGF Model. Subsequent analyses (e.g., within FLOW-AF) suggest that EGFC can be a critical determinant of overall ablation success in advanced substrates. Hence, the model's performance may vary across phenotypes that were not well-represented in the initial training data.

The retrospective nature of the training cohort means that follow-up protocols (e.g., frequency of ECG monitoring or Holter recordings) might not have been fully uniform. Although the FLOW-AF trial provides prospective validation, the sample size there remains modest, warranting confirmation in larger, multicenter studies.

The results primarily represent patients with persistent AF recruited from European centers. Ongoing prospective studies, including the RESOLVE-AF trial (NCT05883631), will further clarify how the optimized EGF Model performs across diverse patient populations and different healthcare systems. To date, evidence is insufficient regarding whether source behavior identified by EGF mapping in patients with paroxysmal AF, mapped during induced or spontaneous episodes, differs significantly from persistent AF. Although post-ablation recurrence following successful pulmonary vein isolation is typically lower in paroxysmal AF patients compared to those with persistent AF, future studies are anticipated to include paroxysmal cohorts.

### Future directions

Our findings suggest that training the hyperparameters of a mechanistic model, one that captures divergent wavefront patterns in AF, can successfully identify ablation targets using a relatively small dataset of only 199 patients. Ongoing refinements to the model and additional prospective trials aim to further enhance this performance. In particular, the upcoming RESOLVE-AF trial—a large, prospective, multinational study—will evaluate the impact of the next iteration of model-based EGF-guided ablation on patient outcomes. This investigation will also help delineate how best to integrate EGF mapping into clinical practice and confirm the potential for improved long-term success in patients with persistent or long-standing persistent AF.

## Conclusion

The EGF mapping algorithm utilizes low-density multielectrode basket electrograms to detect divergent activation wavefronts. By employing a machine-learning approach, the algorithm is trained to detect clinically significant AF sources whose ablation improves clinical outcomes.

## Data Availability

The processed data from this study is owned by Cortex Inc., which, during the review process, became a wholly owned subsidiary of Boston Scientific Corporation. The datasets represent a significant part of the intellectual property acquired in this transaction and are now proprietary to Boston Scientific Corporation. The original anonymized patient data remains under the ownership of the respective clinical centers in Europe. Requests to access the data should be directed to pruppersberg@cortexep.com.
