# Supplementary material for: Statistics and behavior of clinically significant extra-pulmonary vein atrial fibrillation sources: machine-learning-enhanced electrographic flow mapping in persistent atrial fibrillation
Source: Front Cardiovasc Med. 2025 Aug 26;12:1517484. doi: 10.3389/fcvm.2025.1517484 (PMC12417512; doi:10.3389/fcvm.2025.1517484)
Supplement: Supplementary file 1 [file Datasheet1.pdf]

Supplementary Material – Comprehensive Technical Description of the EGF Algorithm

---

1. Theoretical foundations and historical context

Electrographic-Flow (EGF) mapping was conceived to overcome the well-known shortcomings of phase, dominant-frequency and dispersion mapping when used to localise extra-pulmonary-vein atrial-fibrillation (AF) drivers. It treats every unipolar voltage frame recorded by a multielectrode basket as a two-dimensional *intensity field* and reconstructs the continuous velocity vector field that transports those intensities through space and time. The approach borrows directly from the Horn–Schunck optical-flow formalism originally described for computer vision (30). By enforcing **brightness constancy** (the local depolarisation spike is conserved while it moves) and **spatial smoothness** (neighbouring vectors cannot vary arbitrarily), EGF generates a coherent panorama of organised conduction even in apparently chaotic AF (16). Early clinical reports demonstrated that this flow field reliably distinguishes
(i) *centrifugal* wavefronts that radiate from an active focal/rotational driver from
(ii) *centripetal* swirl patterns that merely circulate round anatomical or fibrotic obstacles (17).
In a 25-patient head-to-head series, Bellmann *et al.* confirmed most FIRM rotors but showed that ≈40 % of those rotors displayed **no centrifugal out-flow** and were therefore passive (17). Subsequent work linked the **vector-magnitude** and **temporal persistence** of each source to its clinical relevance: stable, high-velocity sources were the ones whose ablation normalised the flow field and improved outcome (18). These insights underpin the prevalence/activity thresholds adopted in the present model.

---

2. Detailed mathematical formulation

After QRS subtraction and 0.5–150 Hz band-pass filtering, each 1-min, 1-kHz basket recording is down-sampled into N_f frames of Δt ≈ 19 ms. Let $I(x, y, t)$ denote the interpolated voltage at Cartesian basket coordinates (x, y) and time t. The **optical-flow constraint**

$$I_x u + I_y v + I_t = 0$$

is solved together with a quadratic smoothness penalty

$$E(u, v) = \iint \; (I_x u + I_y v + I_t)^2 + \alpha^2(\|\nabla u\|^2 + \|\nabla v\|^2)\, dx\, dy$$

by iterative Horn–Schunck updates; α (regularisation) is one of the 24 trainable hyper-parameters. The resulting dense vector field $\mathbf{V}(x, y, t) = (u, v)$ is integrated over contiguous, 2-s, 50 %-overlapping segments to produce a **segment flow map**. Within each segment the divergence

$$\operatorname{div} \mathbf{V} = \frac{\partial u}{\partial x} + \frac{\partial v}{\partial y}$$

is thresholded; pixels exceeding the divergence cut-off are agglomerated with a density-based clustering routine to yield *candidate sources*. Segment-wise source centroids are then accumulated over

the full 60-s episode to create a **Summary Map** whose colour scale encodes *prevalence* (percentage of segments in which the source is present). Wavefront evolution from the first 0.4 s to the steady 60-s map—illustrated in Fig. 1B—shows how early, weak singularities resolve into one or more dominant sources as further frames are assimilated.

3. Hyper-parameter set and optimisation strategy

**Category (examples)**
Signal conditioning (filter corners, QRS template width, EGM-amp normalisation)
Optical-flow tuning (α, iteration limit, multiframe window length)
Interpolation / mesh parameters (surface tension, biharmonic weight)
Source extraction (divergence cut-off, merge radius, minimum segment prevalence)
Integration & reporting (segment length = 2 s, overlap, Summary-Map bin size)
Clinical threshold (activity cut-off = 26.5 %)

All 24 parameters were optimised on 199 patients using five-fold random cross-validation and 12-month recurrence as ground-truth. The search began with 110 low-noise cases to tune *specificity*, then expanded to the remaining 89 to balance *sensitivity*. The final set delivered 75 % accuracy / 98 % specificity for predicting recurrence by the presence of a ≥26.5 %-active source (see Fig. 1A). Parameter stability was confirmed across folds (SD of optimal α < 4 %).

4. Activity, prevalence and cycle-length metrics

Prevalence (P) is the fraction of 2-s segments in which a source cluster re-appears; sources with $P \geq 20\%$ are termed **dominant**. *Activity* (A) rescales each segment's instantaneous prevalence by the **segment maximum** to offset intermittent loss of contact and is more predictive of outcome. The empirically derived cut-off $A = 26.5\%$ defines a **clinically significant source**.

Cycle-length (CL) analysis is performed on the same 60-s episodes: autocorrelation detects local activation intervals per electrode; channels with correlation < 0.6 or near-field-to-far-field ratio < 0.7 are excluded. Remaining CLs are averaged every 2 s; their spatial SD quantifies dispersion. As shown in Fig. 2C, activation of a dominant source halves the spatial CL SD irrespective of whether mean CL shortens or lengthens, confirming an entrainment effect (22).

5. Position relative to competing technologies

Unlike phase-mapping, which can mistake passive rotations for drivers, EGF's vector divergence makes this distinction explicit (17). Dominant-frequency (DF) maxima often fail to align with EGF-verified sources (36), highlighting the added directional information in flow fields. Spatiotemporal-dispersion ablation terminates AF in many patients (12) but requires point-by-point annotation; EGF offers an automated chamber-wide alternative and, in the FLOW-AF randomised trial, provided a 51 % absolute improvement over PVI-only redo ablation (22).

6. Algorithmic implementation and computational notes

Spline interpolation uses a Green-biharmonic kernel on a 200 × 200 grid, giving ≈1-mm virtual resolution while preserving exact electrode voltages. Optical-flow iterations are GPU-parallelised; one 1-min episode (≈3 000 frames) processes in ≈35 s on a modern workstation, permitting near-real-time display during ablation. Vector fields are down-sampled to 20 × 20 when rendered to minimise visual clutter; prevalence heat-maps retain full resolution.

7. Key validation milestones

- Acute human proof-of-concept (25 pts): active vs. passive rotor discrimination (17).
- Velocity / persistence correlation with successful ablation (18).
- Minute-to-minute and 3-month inter-procedural reproducibility (23).
- Randomised controlled outcome benefit (FLOW-AF, 85 pts) (22).
- Experimental ground-truth localisation in paced AF model (36).

Together these data establish EGF as a rigorously validated, mechanism-based driver-mapping technology suitable for routine clinical use in persistent AF.

Numbers in parentheses refer to the reference list in the main manuscript.
